# Prevalence of and Risk Factors Associated With Nonfatal Overdose Among Veterans Who Have Experienced Homelessness

**DOI:** 10.1001/jamanetworkopen.2020.1190

**Published:** 2020-03-17

**Authors:** Kevin R. Riggs, April E. Hoge, Aerin J. DeRussy, Ann Elizabeth Montgomery, Sally K. Holmes, Erika L. Austin, David E. Pollio, Young-il Kim, Allyson L. Varley, Lillian Gelberg, Sonya E. Gabrielian, John R. Blosnich, Jessica Merlin, Adi V. Gundlapalli, Audrey L. Jones, Adam J. Gordon, Stefan G. Kertesz

**Affiliations:** 1 Birmingham VA Medical Center, Birmingham, Alabama; 2 University of Alabama at Birmingham School of Medicine, Birmingham; 3 University of Alabama at Birmingham School of Public Health, Birmingham; 4 VA Greater Los Angeles Health Care System, Los Angeles, California; 5 David Geffen School of Medicine at University of California, Los Angeles, Los Angeles; 6 Pittsburgh VA Medical Center, Pittsburgh, Pennsylvania; 7 University of Utah School of Medicine, Salt Lake City; 8 Centers for Disease Control and Prevention, Atlanta, Georgia; 9 VA Salt Lake City Health Care System, Salt Lake City, Utah

## Abstract

**Importance:**

Individuals with a history of homelessness are at increased risk for drug or alcohol overdose, although the proportion who have had recent nonfatal overdose is unknown. Understanding risk factors associated with nonfatal overdose could guide efforts to prevent fatal overdose.

**Objectives:**

To determine the prevalence of recent overdose and the individual contributions of drugs and alcohol to overdose and to identify characteristics associated with overdose among veterans who have experienced homelessness.

**Design, Setting, and Participants:**

This survey study was conducted from November 15, 2017, to October 1, 2018, via mailed surveys with telephone follow-up for nonrespondents. Eligible participants were selected from the records of 26 US Department of Veterans Affairs medical centers and included veterans who had received primary care at 1 of these Veterans Affairs medical centers and had a history of experiencing homelessness according to administrative data. Preliminary analyses were conducted in October 2018, and final analyses were conducted in January 2020.

**Main Outcomes and Measures:**

Self-report of overdose (such that emergent medical care was obtained) in the previous 3 years and substances used during the most recent overdose. All percentages are weighted according to propensity to respond to the survey, modeled from clinical characteristics obtained in electronic health records.

**Results:**

A total of 5766 veterans completed the survey (completion rate, 40.2%), and data on overdose were available for 5694 veterans. After adjusting for the propensity to respond to the survey, the mean (SD) age was 56.4 (18.3) years; 5100 veterans (91.6%) were men, 2225 veterans (38.1%) were black, and 2345 veterans (40.7%) were white. A total of 379 veterans (7.4%) reported any overdose during the past 3 years; 228 veterans (4.6%) reported overdose involving drugs, including 83 veterans (1.7%) who reported overdose involving opioids. Overdose involving alcohol was reported by 192 veterans (3.7%). In multivariable analyses, white race (odds ratio, 2.44 [95% CI, 2.00-2.98]), self-reporting a drug problem (odds ratio, 1.66 [95% CI, 1.39-1.98]) or alcohol problem (odds ratio, 2.54 [95% CI, 2.16-2.99]), and having witnessed someone else overdose (odds ratio, 2.34 [95% CI, 1.98-2.76]) were associated with increased risk of overdose.

**Conclusions and Relevance:**

These findings suggest that nonfatal overdose is relatively common among veterans who have experienced homelessness. While overdose involving alcohol was more common than any specific drug, 1.7% of veterans reported overdose involving opioids. Improving access to addiction treatment for veterans who are experiencing homelessness or who are recently housed, especially for those who have experienced or witnessed overdose, could help to protect this population.

## Key Points

**Question:**

How common is nonfatal overdose among veterans who have experienced homelessness, and what are the risk factors and substances involved in overdoses?

**Findings:**

In this survey study including 5766 veterans nationwide who have experienced homelessness, 7.4% of veterans reported an overdose in the previous 3 years. Among veterans reporting overdose, alcohol was the most commonly involved substance.

**Meaning:**

These findings suggest that nonfatal overdose is a relatively common issue among veterans who have experienced homelessness and one that warrants additional attention.

## Teaser

This survey study examines the prevalence of and risk factors associated with nonfatal drug or alcohol overdose among veterans who have experienced homelessness.

## Introduction

Drug overdose accounts for approximately 70 000 deaths annually in the United States,^1^ and nonfatal overdose accounts for more than 500 000 emergency department visits each year.^2^ However, overdose risk varies substantially among individuals and across populations. Individuals who have experienced homelessness, including approximately 4% of Americans,^3^ are at increased risk. Overdose is one of the most common causes of death for younger individuals experiencing homelessness, with opioids implicated in most cases.^4^ However, the prevalence of nonfatal overdose among this population is unknown. Understanding the prevalence of and risk factors associated with nonfatal overdose among individuals experiencing homelessness could inform risk mitigation interventions at approximately 300 federally funded Health Care for the Homeless programs in community health centers,^5^ approximately 55 patient-centered medical homes in the US Department of Veterans Affairs (VA) that are tailored for individuals experiencing homelessness (known as *Homeless Patient Aligned Care Teams* [H-PACTs]),^6^ and other clinical settings that care for individuals experiencing homelessness.

Previous studies on risk factors associated with overdose in individuals experiencing homelessness are limited by several factors. One challenge is that overdose data reported to the National Center on Health Statistics^1,7^ do not include housing status. Additionally, studies from single institutions or geographic regions have limited generalizability owing to region-specific variables, including drug supply contamination.^4^ To our knowledge, there has not been a nationally representative study of overdose prevalence among individuals experiencing homelessness.

As part of a study to examine the association of primary care service design with care experience among patients experiencing homelessness, we administered a survey that included questions about overdose to veterans who have experienced homelessness. This study describes the prevalence of self-reported overdose involving drugs or alcohol in the preceding 3 years, the reported contribution of various drugs and alcohol to overdose, and the demographic, social, and health characteristics associated with overdose in this population.

## Methods

This survey study provides analysis of responses to a national survey of veterans who have experienced homelessness who were receiving primary care at 26 VA medical centers across the United States. The primary aim of the overall study was to compare care experiences across primary care models among veterans who have experienced homelessness. This report represents a secondary aim of that study. This study was approved by the VA’s Central Institutional Review Board. Participants were informed that by answering the survey they were agreeing to participate in the research study, so signed informed consent was not obtained.

### Sample

Eligibility was determined from the VA’s national electronic medical records in November 15, 2017. Eligible veterans were those who had evidence of having experienced homelessness, based on at least 1 *International Classification of Diseases, Ninth Revision, Clinical Modification*^8^ or *International Statistical Classification of Diseases, Tenth Revision, Clinical Modification*^9^ diagnosis of homelessness or VA-specific administrative indicators of receipt of VA homeless services,^10^ in the preceding 30 months. Eligibility also required use of the VA’s primary care services at 1 of 26 VA medical centers, including a single active panel assignment and 2 or more visits to a clinic with an administrative code indicating primary care at the same study site in the preceding 24 months. Included VA medical centers were those with the largest H-PACTs. We randomly selected a subsample of eligible veterans from each study site based on initial power calculations and expected survey response rate of 40%, with the goal of a final sample consisting of two-thirds from H-PACTs and one-third from nontailored mainstream primary care clinics. We later excluded participants if they had no contact information from VA or other records or had died prior to survey initiation. To compare survey respondents and nonrespondents, we retrieved demographic and clinical information from VA medical records in the 2 years preceding recruitment.

### Recruitment

A professional survey organization, Strategic Research Group of Columbus, Ohio, was contracted for recruitment and survey collection. Contact information from VA records was verified, updated, and standardized through a commercial address verification and update product (MelissaData). For instances in which both the MelissaData- and VA-provided addresses resulted in returned mail, Strategic Research Group searched for individual addresses using Whitepages Premium, an online service for finding individuals and verifying addresses. Residential and institutional addresses, including homeless shelters or offices, were used. Participants were recruited in 4 successive 4- to 6-week waves of approximately 3658 individuals each starting in March 2018. Each wave included 4 mailings: an introductory letter, a survey packet with $1 incentive enclosed, a postcard reminder, and, for nonrespondents, a second survey packet. Strategic Research Group called all nonrespondents up to 5 times during a 4-week window with an option to complete the survey by telephone or to provide a new address to receive the survey by mail. A debit card of $10 value was issued after survey completion. We continued to accept returned surveys through October 1, 2018.

### Survey Measures

In the absence of validated survey questions for experiencing overdose, we devised 2 survey items for this study based on similar questions published in the literature^11,12,13^ and on consultation with national experts. Experiencing overdose was based on the question “In the last 3 years, have you had an overdose where you needed to go to the emergency room or get medical care right away?” with a follow-up question asking what substances were involved (eg, alcohol, heroin, fentanyl) (eFigure in the Supplement). Additionally, we devised a similarly worded question to assess whether participants had witnessed someone else overdose (“In the last 3 years, have you seen another person have an overdose where they needed to go to the emergency room or get medical care right away?”) The substances involved with witnessed overdoses were not queried.

In addition to self-reported demographic characteristics, including age, race, ethnicity, and current housing status, other measures obtained via survey included self-report of drug or alcohol problems, based on the validated 2-Item Conjoint Scale,^14^ which assesses having used alcohol or drugs “more than you meant to” and having “felt you wanted to or needed to cut down” in the past 12 months. The survey also included questions on general health status, presence of 8 common medical conditions,^15,16^ chronic pain from a validated 2-item screener,^17,18^ current pain severity (range, 0-10, with higher score indicating more intense pain), severe chronic pain (a binary indicator based on screening positive for chronic pain on the 2-item screen and rating current pain ≥7), and psychological distress symptoms in the past 2 weeks based on the Colorado Mental Health Symptoms Index^19^ as modified and validated for homeless populations (range, 0-24, with higher score indicating more distress and high distress defined as a score >10).^20^ A 6-item indicator of social support was devised from combining 4 items from the National Institutes of Health Patient-Reported Outcomes Measurement Information Set emotional support scale^21^ with a single item on perceived isolation and a tangible support indicator based on ability to borrow $20 from others (range, 0-6, with higher score indicating more support).

### Statistical Analysis

In the initial step of our analysis, we compared survey respondents and nonrespondents on sociodemographic and clinical variables. We used independent sample *t* tests to test for group differences on continuous variables and χ^2^ tests for differences on categorical variables. Next, we constructed a variable for the propensity to respond using the administrative data that were available for each participant who was invited to participate, which included demographic variables (ie, age, sex, race, and marital status), medical conditions (ie, alcohol use disorder, drug use disorder, posttraumatic stress disorder, psychotic disorders, and number of Elixhauser comorbidities), health care utilization information (ie, number of primary care visits, mental health visits, emergency department visits, hospitalizations, and number of administrative service codes related to homelessness), and primary care type (ie, H-PACT vs mainstream). Responses were weighted by the inverse of response propensity (1 / propensity) so that respondents with lower overall propensity to respond were given greater weight, and vice versa. We then compared individuals who experienced any overdose with those who did not.

Finally, we used multivariable logistic regression models to explore which factors were associated with reporting any overdose, overdose involving drugs, and overdose involving alcohol. The intent of these models was explanatory and illustrative rather than predictive. Covariates included sociodemographic and clinical variables shown to be associated with overdose in prior studies: age, race, psychological distress, current homelessness, and medical comorbidities.^22,23,24^ We also included variables associated with homelessness and adverse health outcomes that we hypothesized would be associated with overdose in individuals experiencing homelessness: severe chronic pain^25,26^ and social support.^27,28^ The multivariable analyses excluded individuals with missing data on 1 or more study variables. These models controlled for the identity of the VA medical center through application of a random effects term. *P* values were 2-sided and considered statistically significant at less than .05. Analyses were conducted using SAS statistical software version 9.4 (SAS Institute). Preliminary analyses were conducted in October 2018, and final analyses were conducted in January 2020.

## Results

### Respondents and Nonrespondents

Among 85 719 eligible veterans who had experienced homelessness, we attempted to recruit 14 656 veterans. We excluded 22 veterans who had no contact information and 294 veterans who died prior to survey initiation. Therefore, 14 340 eligible veterans who had experienced homelessness were invited to participate in the survey, and 5766 veterans (40.2%) returned at least partially completed surveys. Characteristics of survey respondents and nonrespondents are presented in the eTable in the Supplement. Of note, response rates were lower among veterans who had experienced homelessness who received care in H-PACT clinics (37.3%) compared with those receiving mainstream primary care (45.2%), for veterans aged 18 to 49 years (27.2%) compared with those aged 50 to 65 years (44.8%) or older than 65 years (46.2%), and for veterans with 2 to 6 primary care visits in the preceding 24 months (33.4%) compared with those with 6 to 11 visits (40.5%) or more than 11 visits (46.4%).

### Unadjusted Associations With Experiencing Nonfatal Overdose

Among 5694 veterans included in the overdose analyses, the mean (SD) age was 56.4 (18.3) years; 5100 veterans (91.6%) were men, 2225 veterans (38.1%) were black, and 2345 veterans (40.7%) were white. Overdose in the previous 3 years was reported by 379 veterans (7.4%), and witnessing someone else overdose was reported by 873 veterans (16.2%). Characteristics of veterans with and without overdose are presented in Table 1. Veterans reporting an overdose, compared with those without overdose, were younger (mean [SD] age, 53.5 [19.3] years vs 56.6 [18.2] years), were more likely to be white (204 veterans [54.2%] vs 2141 veterans [39.6%]; *P* < .001), were more likely to be experiencing homelessness at the time of the survey (77 veterans [21.6%] veterans vs 669 veterans [13.9%]; *P* < .001), were more likely to be receiving medication for mental health (212 veterans [57.8%] vs 1735 veterans [34.3%]; *P* < .001), had higher psychological distress scores (mean [SD] score, 11.1 [11.1] vs 7.2 [10.3]; *P* < .001), and were more likely to report a problem involving alcohol (211 veterans [57.1%] vs 1406 veterans [28.0%]; *P* < .001) or drugs (117 veterans [34.3%] vs 658 veterans [13.7%]; *P* < .001). Veterans who reported an overdose were significantly more likely to have witnessed an overdose than those who did not report an overdose (140 veterans [38.5%] vs 728 veterans [14.4%]; *P* < .001).

**Table 1.** Characteristics of Veterans Who Have Experienced Homelessness Stratified by Recent Overdose

Characteristic	No. (weighted %)^a^		*P* Value
	Overdose (n = 379)	No overdose (n = 5315)	
Age, mean (SD), y	53.5 (19.3)	56.6 (18.2)	
Men^b^	345 (92.7)	4755 (91.5)	.18
Race			
White	204 (54.2)	2141 (39.6)	<.001
Black	96 (23.2)	2129 (39.3)	
Other	79 (22.5)	1045 (21.1)	
Hispanic ethnicity^c^	53 (15.2)	540 (11.3)	<.001
Current homelessness^d^	77 (21.6)	669 (13.9)	<.001
Receiving medication for mental health^e^	212 (57.8)	1735 (34.3)	<.001
Psychological distress score, mean (SD)^f^^,^^g^	11.1 (11.1)	7.2 (10.3)	<.001
High psychological distress^f^^,^^h^	195 (57.0)	1518 (32.7)	<.001
Self-reported comorbidities, mean (SD), No.^i^	1.8 (2.7)	1.7 (2.3)	.70
Low self-rated health^j^	300 (82.9)	4225 (81.7)	.35
Alcohol problem^k^	211 (57.1)	1406 (28.0)	<.001
Drug problem^k^	117 (34.3)	658 (13.7)	<.001
Severe chronic pain	158 (41.1)	2003 (37.1)	.01
Social support score, mean (SD)^l^	3.8 (3.4)	4.3 (3.1)	<.001
Witnessed overdose^m^	140 (38.5)	728 (14.4)	<.001
Received H-PACT primary care	245 (68.5)	3106 (62.6)	<.001

Abbreviations: H-PACT, Homeless Patient Aligned Care Team.

^a^ Percentages are weighted for propensity to respond to the survey.

^b^ Missing data for 91 individuals.

^c^ Missing data for 128 individuals.

^d^ Missing data for 178 individuals.

^e^ Missing data for 71 individuals.

^f^ Missing data for 432 individuals.

^g^ Psychological distress score is based on Colorado Mental Health Symptoms Index as modified and validated for homeless populations (range, 0-24, with higher scores indicating more psychological distress).

^h^ Defined as psyschological distress score greater than 10.

^i^ Includes diabetes, hypertension or high blood pressure, coronary heart disease, myocardial infarction, cerebrovascular disease or stroke, asthma, emphysema, and arthritis. Missing data for 66 individuals.

^j^ Missing data for 214 individuals.

^k^ Missing data for 68 individuals.

^l^ Calculated by combining 4 items from the National Institutes of Health Patient-Reported Outcomes Measurement Information Set emotional support scale (range, 0-6, with higher score indicating more support). Missing data for 324 individuals.

^m^ Missing data for 20 individuals.

### Substances Involved With Nonfatal Overdose

Alcohol was the most common substance involved in a recent overdose, reported by 192 veterans (3.7%) (Table 2). Overdose involving any drug was reported by 228 veterans (4.6%). Specifically, overdose involving any opioid was reported by 83 veterans (1.7%), with heroin or fentanyl being the most frequently reported opioid (53 veterans [1.2%]). The category other (representing any drug other than those named on the survey form) was endorsed by 103 veterans (2.1%), while 16 veterans (0.3%) reported overdose but did not specify any involved substance.

**Table 2.** Substances Involved in Most Recent Overdose Among Veterans Who Have Experienced Homelessness Reporting Overdose

Substance	No. (weighted %)^a^
Alcohol	192 (3.7)
Any drug	228 (4.6)
Opioids	83 (1.7)
Heroin or fentanyl	53 (1.2)
Analgesics	35 (0.6)
Methadone	8 (0.2)
Sedatives	23 (0.5)
Gabapentin or pregabalin	22 (0.4)
Cocaine	55 (1.1)
Other drug	103 (2.1)
No substance specified	16 (0.3)

^a^ Individuals could endorse more than 1 substance, so the total sums to greater than 379.

### Adjusted Associations With Experiencing Any Overdose, Overdose Involving Alcohol, and Overdose Involving Drugs

Results of the multivariable models for experiencing any overdose, overdose involving alcohol, and overdose involving drugs are presented in Table 3. Characteristics associated with any overdose included white race (odds ratio [OR], 2.44 [95% CI, 2.00-2.98]), current homelessness (OR, 1.35 [95% CI, 1.11-1.63]), receipt of a psychiatric medication (OR, 2.05 [95% CI, 1.75-2.41]), elevated psychological distress (OR per 1-point increase, 1.05 [95% CI, 1.04-1.06]), self-reporting a drug problem (OR, 1.66 [95% CI, 1.39-1.98]) or alcohol problem (OR, 2.54 [95% CI, 2.16-2.99]), and having witnessed someone else overdose (OR, 2.34 [95% CI, 1.98-2.76]). When overdoses involving alcohol or drugs were modeled separately, similar variables emerged as significant, with several notable exceptions. Veterans younger than 50 years were more likely than older veterans to report drug overdoses (OR, 1.78 [95% CI, 1.42-2.22]). Veterans who reported drug problems were less likely than veterans who did not report drug problems to report an overdose involving alcohol (OR, 0.47 [95% CI, 0.36-0.61]), and veterans who reported alcohol problems were less likely than veterans who did not report alcohol problems to report an overdose involving drugs (OR, 0.74 [95% CI, 0.59-0.91]).

**Table 3.** Adjusted Associations of Respondent Characteristics With Reporting Any Overdose, Drug-Related Overdose, and Alcohol-Related Overdose

Characteristic	Overdose, odds ratio (95% CI)		
	Any	Drug-related	Alcohol-related
Age <50 y	1.19 (0.99-1.43)	1.78 (1.42-2.22)	0.87 (0.68-1.12)
Women	0.87 (0.65-1.17)	1.27 (0.92-1.77)	0.68 (0.43-1.09)
Race			
Black	1 [Reference]	1 [Reference]	1 [Reference]
White	2.44 (2.00-2.98)	1.96 (1.53-2.52)	3.54 (2.64-4.74)
All other	1.48 (1.16-1.90)	1.55 (1.15-2.08)	1.72 (1.20-2.48)
Hispanic ethnicity	1.11 (0.88-1.41)	1.08 (0.81-1.45)	1.41 (1.03-1.93)
Current homelessness	1.35 (1.11-1.63)	1.30 (1.03-1.65)	1.22 (0.93-1.61)
Receiving medication for mental health	2.05 (1.75-2.41)	1.91 (1.56-2.33)	2.11 (1.68-2.65)
Psychological distress score, per 1-unit increase^a^	1.05 (1.04-1.06)	1.05 (1.03-1.07)	1.05 (1.03-1.07)
Self-reported comorbidities, per 1-unit increase^b^	0.98 (0.93-1.04)	1.08 (1.00-1.15)	0.87 (0.80-0.94)
Low self-rated health	0.90 (0.73-1.10)	0.95 (0.73-1.24)	1.11 (0.82-1.51)
Drug problem in last 12 mo	1.66 (1.39-1.98)	5.14 (4.14-6.39)	0.47 (0.36-0.61)
Alcohol problem in last 12 mo	2.54 (2.16-2.99)	0.74 (0.59-0.91)	12.85 (9.79-16.85)
Severe chronic pain	0.81 (0.69-0.96)	0.87 (0.70-1.08)	1.00 (0.79-1.27)
Social support, per 1-unit increase^c^	1.02 (0.98-1.06)	1.06 (1.01-1.11)	1.03 (0.97-1.09)
Witnessed overdose	2.34 (1.98-2.76)	1.98 (1.61-2.43)	2.71 (2.16-3.40)
Receiving H-PACT primary care	1.09 (0.92-1.28)	1.12 (0.91-1.38)	1.21 (0.96-1.53)

Abbreviations: H-PACT, Homeless Patient Aligned Care Team.

^a^ Psychological distress score is based on Colorado Mental Health Symptoms Index as modified and validated for homeless populations (range, 0-24, with higher scores indicating more psychological distress).

^b^ Included diabetes, hypertension or high blood pressure, coronary heart disease, myocardial infarction, cerebrovascular disease or stroke, asthma, emphysema, and arthritis.

^c^ Calculated by combining 4 items from the National Institutes of Health Patient-Reported Outcomes Measurement Information Set emotional support scale (range, 0-6, with higher score indicating more support).

## Discussion

In this large survey study of veterans who have experienced homelessness, 7.4% reported an overdose requiring urgent medical attention in the past 3 years, and 16.2% had witnessed someone else overdose. Witnessing overdose was significantly associated with personal experience of overdose. Alcohol was the predominant substance reported in overdose, although 1.7% of veterans reported overdose involving opioids.

One potentially actionable finding from this study is that alcohol was the most common substance reported with overdose, nearly as common as all drugs combined and more than 2-fold as common as opioids. Previous studies have shown that alcohol, cannabis, and cocaine are the most widely used substances among individuals experiencing homelessness.^29,30^ Alcohol overdose, or acute intoxication, is a frequent cause for emergency department visits.^31^ Although alcohol rarely appears as the sole toxin in fatal overdoses,^32^ heavy alcohol use has a substantial negative effect on long-term morbidity and mortality.^33^ Alcohol use disorder is also treatable with approved medications and interventions, such as motivational interviewing and cognitive behavioral therapy.^34^ Despite this, data show that most individuals with alcohol use disorder do not receive treatment.^35^

The second most common overdose cause was opioids. Opioid overdose in this sample (3-year prevalence, 1.7%) was higher than for the general population^2^ and comparable with an analysis of patients with Medicaid insurance who had received at least 1 opioid prescription, in which 0.5% of patients per year required medical services for overdose involving opioids.^36^ A 2018 study using Medicaid data^37^ found that individuals who survived nonfatal opioid overdose were at 24-fold higher risk of death compared with the general population, with drug overdose, respiratory illness, HIV, and suicide all contributing to mortality. For health systems that serve individuals experiencing homelessness, a patient’s report of recent overdose signals elevated medical risk and may even merit standardized screening or offer of additional services.

Additionally, maximizing access to the opioid overdose reversal agent naloxone is advisable.^38^ However, since pharmacies are required to dispense naloxone to a named individual rather than a facility, this can be difficult to accomplish for homeless shelters. Finally, opioid overdose could also be prevented through medication therapy for opioid use disorder,^39^ yet focused efforts to assure delivery of treatment for opioid use disorder in this population are uncommon.^40^

The findings of this study may suggest additional targets for clinical and policy intervention. The association of witnessing overdose with experiencing overdose suggests that efforts at risk mitigation should take into account patients’ social networks. Although relevant to substance use,^41^ few studies explore social networks among veterans who have experienced homelessness, a unique population whose relationships may be shaped by history of military service and unpredictability of housing. Peer-based programs have been applied widely in VA medical centers,^42^ including with veterans who have experienced homelessness.^43^ Peer-based strategies that focus on homelessness may be an opportunity for tailored assistance, potentially tapping into the experience and wisdom of veterans who have experienced homelessness in reaching their peers.

Given the prevalence of high emotional distress in individuals who have experienced overdose, enhanced mental health services could mitigate some risk for individuals residing on the streets (also known as *sleeping rough*), in homeless shelters, or newly in housing. This could be advanced through outreach, colocation of mental health and primary care services, or enhancing the capacity of primary care clinicians to deliver mental health care. Motivational interviewing to address substance use has been shown to be effective in homeless populations,^44,45,46,47^ provided there are viable addiction treatment options available.^48,49^ While federal grants have expanded care for opioid use disorder, block grants from the Substance Abuse and Mental Health Services Administration, such as those that would assist with alcohol use disorder, have received flat funding since 2014 and fallen relative to inflation.^50^

### Limitations

This study has some limitations. First, the cross-sectional nature of the survey prevents causal inference. Current homelessness, for example, was associated with overdose, but it is possible that having an overdose or serious addiction contributed to loss of residence. Second, the response rate was only 40.2%, which would be considered suboptimal under some circumstances. However, this response rate is comparable to that obtained in an evaluation of national VA primary care (47%),^51^ and roughly 2-fold that obtained by VA’s primary care survey of veterans who have experienced homelessness.^6^ Furthermore, since we had access to rich data from VA records to compare respondents and nonrespondents, we were able to weight our analyses by the propensity to respond, which at least somewhat reduces the risk of nonresponse bias. It is also important to highlight that there have been few recent efforts to survey homeless-experienced populations on a national basis,^52,53^ so this study likely represents the best available data to study these questions in this population. Third, generalizability may be limited because our sample consisted only of veterans who accessed VA services and who were reachable through mail or telephone. In general, the VA updates a mailing address with every visit, and a 2014 report^54^ found that 89% of people experiencing homelessness who use VA services had mobile telephones. We speculate that veterans who have experienced homelessness and who are lacking both a mailing address and a telephone number could be at higher risk of overdose than individuals included in this study. Fourth, our study relies on self-reported survey data, and the validity of self-report of overdose is uncertain. However, many factors associated with overdose risk in this study are consonant with prior studies that used medical records, including substance use disorders,^22,23,24^ race,^1,55^ psychological disorders,^23,56^ and having witnessed others overdose.^57^ Fifth, the survey did not include an option for methamphetamine, which could have contributed to the other substance category, and merits specific queries in future research.

## Conclusions

This study, the largest survey of veterans who have experienced homelessness to date, to our knowledge, found that recent nonfatal overdose was a relatively common issue among veterans who have experienced homelessness, occurring in 7.4% of survey respondents, with alcohol being involved more often than opioids. Improving access to mental health and addiction treatment for veterans who are experiencing homeless or who are recently housed and targeting health and social services for those at increased risk could enhance the safety of this population.

## Notes

Supplement. **eTable.** Characteristics of Survey Respondents and Nonrespondents **eFigure.** Survey Question About Recent Personal Overdose
