# Supplementary material for: Prevalence of and Risk Factors Associated With Nonfatal Overdose Among Veterans Who Have Experienced Homelessness
Source: JAMA Netw Open. 2020 Mar 17;3(3):e201190. doi: 10.1001/jamanetworkopen.2020.1190 (PMC7078753; doi:10.1001/jamanetworkopen.2020.1190)
Supplement: Supplement. — eTable. Characteristics of Survey Respondents and Nonrespondents eFigure. Survey Question About Recent Personal Overdose [file jamanetwopen-3-e201190-s001.pdf]

Supplementary Online Content

Riggs KR, Hoge AE, DeRussy AJ, et al. Prevalence of and risk factors associated with nonfatal overdose among veterans who have experienced homelessness *JAMA Netw Open*. 2020;3(3):e201190. doi:10.1001/jamanetworkopen.2020.1190

eTable. Characteristics of Survey Respondents and Nonrespondents

eFigure. Survey Question About Recent Personal Overdose

This supplementary material has been provided by the authors to give readers additional information about their work.

© 2020 Riggs KR et al. *JAMA Network Open.*

eTable. Characteristics of Survey Respondents and Nonrespondents

Characteristic	Overall	Respondents	Non-Respondents	Response Rate	
	n (col %)	*n* (column %)	*n* (column %)	row %	*P* value
Primary Care Clinic					<0.001
H-PACT	9095 (63.4)	3394 (58.9)	5701 (66.5)	37.3%	
Mainstream	5245 (36.6)	2372 (41.4)	2873 (33.5)	45.2%	
Age					<0.001
18-49	3852 (27.6)	1074 (18.6)	2878 (33.6)	27.2%	
50-65	7746 (54.0)	3472 (60.2)	4274 (49.9)	44.8%	
>65	2642 (18.4)	1220 (21.2)	1422 (16.6)	46.2%	
Sex					0.03
Male	13167 (91.8)	5260 (91.2)	7907 (92.2)	40.0%	
Female	1173 (8.2)	506 (8.8)	667 (7.8)	43.1%	
Race					0.02
White	6724 (46.9)	2734 (47.4)	3990 (46.5)	40.7%	
Black	6183 (43.1)	2507 (43.5)	3676 (42.9)	40.6%	
Other	391 (2.7)	152 (2.6)	239 (2.8)	38.9%	
Missing	1042 (7.3)	373 (6.5)	669 (7.8)	35.8%	
Marital status					<0.001
Married	2097 (14.6)	890 (15.4)	1207 (14.1)	42.4%	
Divorced/separated	6936 (48.4)	2886 (50.1)	4050 (47.2)	41.6%	
Never married/other	5307 (37.0)	1990 (34.5)	3317 (38.7)	37.5%	
Income					0.02
<$5,885	6365 (44.4)	2503 (43.4)	3862 (45.0)	39.3%	
$5,885-$11,770	2725 (19.0)	1157 (20.1)	1568 (18.3)	42.5%	
>$11,770	5250 (36.6)	2106 (36.5)	3144 (36.7)	40.1%	
Elixhauser Index					<0.001
0-2	4877 (34.01)	1763 (30.6)	3114 (36.3)	36.2%	
3-4	4974 (33.4)	1977 (34.3)	2817 (32.9)	41.2%	
>5	4669 (32.6)	2026 (35.1)	2643 (30.8)	43.4%	
Traumatic brain injury	501 (3.5)	178 (3.1)	323 (3.8)	35.5%	0.03
Environmental hazards	49 (0.3)	22 (0.4)	27 (0.3)	44.9%	0.50
Mental Health/Substance Use					
PTSD	4167 (29.1)	1506 (26.1)	2661 (31.0)	36.1%	<0.001
Depression	8126 (56.7)	3236 (56.1)	4890 (57.0)	39.8%	0.28
Anxiety disorder	4028 (28.1)	1609 (27.9)	2419 (28.2)	39.9%	0.69
Psychotic disorder	1951 (13.6)	625 (10.8)	1326 (15.5)	32.0%	<0.001
Alcohol use disorder	5848 (40.8)	2258 (39.2)	3590 (41.9)	38.6%	0.001
Drug use disorder	5496 (38.3)	1999 (34.7)	3497 (40.8)	36.4%	<0.001
Any of above	11481 (80.1)	4503 (78.1)	6978 (81.4)	38.2%	<0.001
Healthcare utilization					
Primary care visits					<0.001
2-5	4477 (31.2)	1497 (26.0)	2980 (34.8)	33.4%	
6-11	5152 (35.93)	2085 (36.2)	3067 (35.8)	40.5%	
>11	4711 (32.9)	2184 (37.9)	2527 (29.5)	46.4%	
Emergency department visits					<0.001
<8	12836 (89.5)	5284 (91.6)	7552 (88.1)	41.2%	
≥8	1504 (10.5)	482 (8.4)	1022 (11.9)	32.1%	
Any Hospitalization	5035 (35.1)	1967 (34.1)	3799 (65.9)	39.1%	0.04
Receipt of VA social services					
Health care for homeless Veterans	9920 (69.2)	3747 (65.0)	6173 (72.0)	37.8%	<0.001
Grant and per-diem voucher	3865 (27.0)	1324 (23.0)	2541 (29.6)	34.3%	<0.001
HUD-VASH	8276 (57.7)	3258 (56.5)	5018 (58.5)	39.4%	0.02
Justice outreach	1381 (9.6)	471 (8.2)	910 (10.6)	34.1%	<0.001

Abbreviations: H-PACT = Homeless Patient Aligned Care Team; HUD-VASH = U.S. Housing and Urban Development – Veterans Affairs Supportive Housing; PTSD – post-traumatic stress disorder

© 2020 Riggs KR et al. *JAMA Network Open.*

eFigure. Survey Question About Recent Personal Overdose

5. In the last 3 years, have you had an overdose where you needed to go to the emergency room or get medical care right away?

- ◯ No
- ◯ Yes ➡ 5a. What were you taking at the time of your most recent overdose? (Select all that apply.)
  - ◯ Alcohol
  - ◯ Heroin or fentanyl not from a doctor
  - ◯ Opioid painkillers like Percocet, Lortab, morphine, oxycodone, hydrocodone, tramadol or others
  - ◯ Methadone
  - ◯ Cocaine
  - ◯ Sedatives like Klonopin, Xanax or Valium
  - ◯ Gabapentin, Lyrica or pregabalin
  - ◯ Other

© 2020 Riggs KR et al. *JAMA Network Open.*
